# The addition of employment support alongside psychological therapy enhances the chance of recovery for clients most at risk of poor clinical outcomes

**DOI:** 10.1017/S1352465823000474

**Published:** 2023-10-23

**Authors:** Graham R. Thew, Ana Popa, Claire Allsop, Elaine Crozier, Josef Landsberg, Samantha Sadler

**Affiliations:** 1 Department of Experimental Psychology, University of Oxford, Oxford, UK; 2 NHS Oxfordshire Talking Therapies, Oxford Health NHS Foundation Trust, Oxford, UK; 3 NHS Buckinghamshire Talking Therapies, Oxford Health NHS Foundation Trust, High Wycombe, UK; 4 Berkshire Healthcare NHS Foundation Trust, Bracknell, UK

**Keywords:** Employment support, IAPT, Latent profile analysis, Mental health outcomes, NHS Talking Therapies for Anxiety and Depression, Recovery

## Abstract

**Background:**

Many people achieve positive outcomes from psychological therapies for anxiety and depression. However, not everyone benefits and some may require additional support. Previous studies have examined the demographic and clinical characteristics of people starting treatment and identified a patient profile that is associated with poor clinical outcomes.

**Aims:**

To examine whether the addition of employment-related support alongside psychological therapy was associated with a greater chance of recovery for clients belonging to this patient profile.

**Method:**

We analysed 302 clients across three services, who were offered employment-related support alongside psychological therapy. The rate of clinical recovery (falling below clinical thresholds on measures of both anxiety and depression) was compared between individuals who accepted the offer and those who declined, while adjusting for potential confounders.

**Results:**

Logistic regression showed that receiving employment support was significantly associated with clinical recovery after controlling for baseline anxiety and depression scores, the number of psychological treatment sessions, and other clinical and demographic variables. The odds of recovery were 2.54 times greater if clients received employment support; 47% of clients who received employment support alongside psychological therapy were classified as recovered, compared with 27% of those receiving psychological therapy only.

**Conclusions:**

Providing employment support alongside therapy may be particularly helpful for clients belonging to this patient profile, who represent approximately 10% of referrals to NHS Talking Therapies for Anxiety and Depression services. Services could consider how to increase the provision and uptake of employment-focused support to enhance clients’ clinical outcomes.

## Introduction

Psychological therapies such as cognitive behaviour therapy have strong empirical support for the treatment of anxiety disorders and depression, and as a result are recommended by many national healthcare bodies (e.g. APA, 2019; Asakura *et al*., 2023; Katzman *et al*., 2014), including the National Institute for Health and Care Excellence (NICE) in the UK (e.g. NICE, 2009, 2013, 2018). However, data from clinical trials and routine practice settings demonstrate that not everyone improves during these treatments. Data from NHS Digital shows that in 2021–2022, 33.1% of those receiving a course of psychological therapy for anxiety or depression did not show a reliable improvement in symptoms. One way to address this problem is to use methods to identify individuals who may be at risk of poorer outcomes from therapy, so that we can adapt, tailor, or enhance the interventions we provide to optimise clinical outcomes.

This personalised approach is more established within medical fields such as cancer care (Jackson and Chester, 2015) and diabetes (Nijpels *et al*., 2019), where it is common practice for patients to be screened for factors associated with poor clinical outcomes and have their treatment tailored based on these predictions. Such approaches have started to be used within mental healthcare, for example by using large datasets of previously treated patients to make predictions about which treatment components are likely to be effective for someone entering treatment, and to support monitoring of their progress and feedback to therapists if this is off-track (Lutz *et al*., 2022). The term patient stratification describes the approach where patients are categorised based on their profile of baseline clinical and demographic characteristics. It has been suggested that identifying such groups may help improve our understanding of why some patients benefit more from treatment than others, and assist in tailoring treatment to enhance outcomes (Saunders *et al*., 2016).

Saunders *et al*. (2016, 2020) used a latent profile analysis approach to examine a range of routinely collected patient characteristics that could be related to outcome in a sample of 16,000 people treated in two services in London that are part of the NHS Talking Therapies for Anxiety and Depression programme (NHS TTad; formerly ‘Improving Access to Psychological Therapies’). These studies aimed to identify subgroups (or ‘latent profiles’) of patients in the dataset who were similar to each other in terms of clusters of demographic and clinical characteristics. Eight distinct latent profiles were identified. Clients in the seventh profile (LP7), which represented 10% of clients, were least likely to recover following treatment, showing an overall recovery rate of only 15–18%, compared with rates up to 74% in the other profiles. Relative to the other profiles, clients in the LP7 category showed the highest average scores for anxiety (mean=18.38, *SD*=2.50 on the Generalised Anxiety Disorder Questionnaire, GAD-7; Spitzer *et al*., 2006) and depression (mean=22.86, *SD*=2.78 on the Patient Health Questionnaire, PHQ-9; Kroenke *et al*., 2001) at the initial assessment. They were also older than the average for clients using these services (mean=42.74 years, *SD*=9.44), and were more likely to be receiving welfare benefits (74%) and to be prescribed medication (73%). Further information on the patient characteristics for all eight profiles is provided in Saunders *et al*. (2016, 2020).

Clients in the LP7 profile are therefore at particular risk of poor clinical outcomes. Initial examination of data from three NHS TTad services in the Thames Valley led to a hypothesis that the addition of employment support alongside psychological therapy could increase the recovery rates of those individuals in LP7, compared with receiving therapy alone. This was because this group were likely to be receiving welfare benefits and may also have been experiencing employment-related difficulties. Employment support is provided in partnership with a growing number of NHS TTad services due to the interplay between employment status and mental health (Department of Work and Pensions, 2019). Employment support services offer help and guidance to service users who require assistance to retain existing employment, to return to work following a period of ill health, or to gain employment, using a variety of tools and training. There is evidence to suggest the provision of employment support alongside psychological therapy can result in additional benefits to wellbeing, confidence, and motivation, as well as reductions in anxiety and depression (Department of Work and Pensions, 2019; Hogarth *et al*., 2013).

This service evaluation project therefore aimed to use data pooled across three services to examine whether for individuals identified to be in the LP7 profile, the addition of employment support alongside psychological therapy was associated with a greater chance of recovery.

## Method

### Procedure

To identify patients who presented in the LP7 group, we used a bespoke algorithm developed by the authors of the original paper (Saunders *et al*., 2016). This latent profiling algorithm was provided in Microsoft Excel, and allowed the service team to enter a set of variables which are used by the algorithm to allocate each patient into the latent profile to which they had the highest probability of membership. The following variables are used by the algorithm: age at referral, gender, ethnicity, medication prescription status, welfare status, and self-reported symptoms of depression, anxiety, phobia, and level of personal and social functioning. These variables are collected routinely for all patients at the initial intake assessment. For further details of the latent profiling algorithm, please contact the corresponding author. During the period May to June 2021, the data leads in each service applied the algorithm to all patients who attended an initial assessment session. Where a client was classified into the LP7 category, this was highlighted to the clinical team, who gave an explanation of employment support and offered this option to the client. Where this offer was accepted, the patient was referred to an employment advisor who contacted the client to arrange an appointment.

### Participants

During the study period, 466 clients were identified as belonging to the LP7 category and were offered employment support alongside their psychological therapy. To be included in the analysis, participants were required to have completed two or more psychological therapy sessions, which is the standard minimum length of a course of treatment for reporting purposes in NHS TTad services (NHS Digital, 2022). Data from 302 participants were analysed, which were drawn from the Berkshire (*n*=90), Buckinghamshire (*n*=101) and Oxfordshire (*n*=111) NHS TTad services. These services provide a range of NICE-recommended psychological therapies for anxiety and depression. The majority of the sample (*n*=202) were female, and the overall mean age was 41.98 (*SD*=11.26). Of the 302 participants analysed, 66 had received at least one session of employment support, and the remaining 236 declined this offer. Those receiving employment support attended a mean of 3.08 employment support sessions (*SD*=2.26), typically provided on a fortnightly basis. Demographic and clinical information on the sample is provided in Table 1. No significant differences on baseline variables were observed between the group who received employment support and the group who did not.

### Measures

Recovery follows a standard definition across all services within the NHS TTad programme. To be classified as being in recovery, a client must have started treatment above the clinical caseness threshold for *either* anxiety (> = 8 on the GAD-7; Spitzer *et al*., 2006) or depression (> = 10 on the PHQ-9; Kroenke *et al*., 2001), and by the end of treatment show scores that are below these thresholds for *both* anxiety and depression. Alternative anxiety disorder specific measures should be used in place of the GAD-7 if clinically appropriate (for further details, see National Collaborating Centre for Mental Health, 2018). The proportion of clients meeting this recovery criterion at the end of treatment is known as the ‘recovery rate’.

### Analysis

All analyses were performed on the combined dataset from the three services. Clients were defined as receiving employment support if they attended at least one employment support session. First, the recovery rates were examined descriptively, comparing participants who accepted employment support with those who declined. Logistic regression was then used to examine whether receiving employment support or not was significantly associated with recovery status. The logistic regression was performed in two steps. First, the employment support variable was tested in isolation. Second, a set of variables to be examined as possible confounds was added to the model. As participants were not randomised to receiving employment support or not, and the analysis was based on an observational sample, the inclusion of possible confounder variables aimed to mitigate the risk that any employment support effects might be spurious. These variables were: baseline scores on the standard NHS TTad measures of depression (PHQ-9; Kroenke *et al*., 2001), anxiety (GAD-7; Spitzer *et al*., 2006), and general functioning (Work and Social Adjustment Scale, WSAS; Mundt *et al*., 2002), the total number of psychological treatment sessions, problem descriptor (i.e. the provisional clinical diagnosis), presence of a long-term health condition, and client demographic variables (gender, ethnicity and age). Statistical assumptions including multi-collinearity were checked and met for the analysis.

## Results

Overall, 31% of this sample of LP7 participants met NHS TTad recovery criteria. Examination of the descriptive data showed that where clients received employment support, the recovery rate was 47% (31/66), but that a lower rate of 27% (64/236) was observed where clients did not receive employment support. Descriptive data for the variables analysed are shown in Table 1.

Results of the logistic regression are shown in Table 2. The first step showed that receiving employment support was significantly associated with recovery in this sample (OR=2.82, 95% CI=1.56, 5.08, *p*<.001). In the second step, possible confounding variables were also added to the model. Results indicated that the total number of psychological treatment sessions was significantly associated with recovery (OR=1.14, 95% CI=1.08, 1.20, *p*<.001) with more sessions linked to a greater chance of recovery. Having a long-term condition was also significant (OR=0.49, 95% CI=0.27, 0.89, *p*=.020), associated with a poorer chance of recovery. In this second step, receiving employment support remained significant after controlling for these other variables (OR=2.54, 95% CI=1.32, 4.89, *p*=.005). The odds ratio indicated that if a client was in the LP7 category and received employment support, their odds of reaching recovery were 2.54 times greater compared with not receiving employment support.

## Discussion

Overall, the findings indicated that providing employment support was associated with a greater chance of recovery for clients in the LP7 category, who are at greatest risk of poor clinical outcomes. This finding remained the case after accounting for various potential confounding variables (baseline severity, number of psychological treatment sessions, problem descriptor, presence of a long-term health condition, and client demographics). The findings also suggest that a greater number of therapy sessions is likely to be helpful for enhancing outcomes for this client group. As the presence of a long-term health condition was associated with poorer clinical outcomes in this group, exploring ways to provide additional psychological support in the management of such physical health conditions may also be beneficial.

These findings are consistent with existing literature that highlights the mental health benefits of employment support (Department of Work and Pensions, 2019; Hogarth *et al*., 2013). This study is one of the first to directly compare the combination of psychological therapy and employment support, to psychological therapy alone. Further studies are needed to fully understand the extent of additional benefits this combination may provide, using robust methodologies such as randomisation where possible. It is notable that the overall recovery rate for LP7 clients in the present study who did not receive employment support (27%) was higher than the 15–18% found by Saunders *et al*. (2016, 2020). While this could reflect improvements in recovery rates over time (the original papers use data from 2008–2018), investigation of the clinical outcomes of LP7 clients across a wider range of services and geographical locations may further our understanding of the extent to which these vary and may provide further indications of how best to support this client group.

The principal limitation of this study is the fact that participants were not randomised to receiving employment support or not. It is possible that those who accepted employment support may have been more engaged overall with the service and could therefore have been more likely to benefit from treatment. Our analyses aimed to mitigate this by controlling for the effect of possible confounding variables and found the employment support effect remained significant, but a randomised design in future would be beneficial, as we cannot rule out the potential influence of other unmeasured confounders in the present design. We examined only a relatively brief time period, which does not allow for exploring longitudinal trends. Only three services were included, which are close geographically and to some extent demographically. Findings may not therefore generalise to all services and analysis of broader samples is recommended. Lastly, this study did not analyse why people might decline employment support, or the content or quality of the employment support provided. It is possible that people decline employment support due to feeling too anxious or depressed to consider employment-related changes, or have concerns about changes to their benefits status, and these potential barriers to accessing support should be examined in future studies. Although the training of employment support staff is standardised, there may have been individual differences in how this was delivered. Future research could review which aspects of employment support may be most helpful.

As outlined above, the findings offer a number of interesting directions for future research in this area that will help to determine the impact of employment support more definitively for this patient group. To facilitate such evaluations, services may wish to consider their current provision and uptake of employment support and whether this can be improved. An ‘outreach’ model, where LP7 cases are identified at a service level and then prompts given to clinicians to offer employment support may be both resource and time heavy but could prove effective. This method cannot guarantee clients will accept the offer of employment support but does allow for a conversation between the assessing clinician and client to consider the relative benefits. It is important therefore for the therapists to have a good understanding of any employment support available and what this involves. An alternative model would be to offer employment support automatically to all individuals who at assessment report being on sick-leave, unemployed, or who self-identify as needing employment-related help. Future work may wish to examine if this approach is acceptable to clients and how client consent and individual preferences should be accommodated.

Overall, the results of this study may offer a promising route to improve the clinical outcomes of those who otherwise may be less likely to benefit from psychological treatment alone.

## Figures and Tables

**Table 1:** Descriptive data for the variables analysed

Variable	Total sample (*n* = 302)	Received employment support (*n* = 66)	Did not receive employment support (*n* = 236)	Test statistic
Mean PHQ-9 baseline score (*SD*)	22.48 (2.72)	21.91 (3.32)	22.64 (2.52)	*t*_300_ = 1.93, *p* = .054
Mean GAD-7 baseline score (*SD*)	18.19 (2.53)	17.80 (2.30)	18.29 (2.58)	*t*_300_ = 1.40, *p* = .164
Mean WSAS baseline score (*SD*)	30.59 (5.32)	31.36 (4.52)	30.38 (5.51)	*t*_300_ = 1.33, *p* = .186
Mean number of psychological therapy sessions (*SD*)	7.83 (5.80)	8.98 (6.44)	7.51 (5.58)	*t*_300_ = 1.83, *p* = .069
Mean age (*SD*)	41.98 (11.26)	43.91 (9.87)	41.44 (11.58)	*t*_300_ = 1.58, *p* = .115
**Long-term condition**				
Yes	140	29	111	*χ*^2^ (1, N = 281) = 0.30, *p* = .587
No	141	33	108	
Missing	21	4	17	
**Problem descriptor**				
Depressive disorders	179	45	134	*χ*^2^ (1,N = 296) = 2.11, *p* = .146
Other	117	21	96	
Missing	6	0	6	
**Gender**				
Female	202	43	159	*χ*^2^ (1,N = 301) = 0.15, *p* = .702
Male	99	23	76	
Missing	1	0	1	
**Ethnicity**				
White backgrounds	220	51	169	*χ*^2^ (1,N = 292) = 0.44, *p* = .508
Other backgrounds	72	14	58	
Missing	10	1	9	

*Notes*: Problem descriptor and Ethnicity categories were collapsed as shown due to small numbers in some categories. PHQ, Patient Health Questionnaire; GAD, Generalised Anxiety Disorder Questionnaire; WSAS, Work and Social Adjustment Scale.

**Table 2:** Results of the Logistic Regression Analysis

Model	Predictor	Estimate	SE	*p*	Odds ratio	95% CI (OR)
1	Intercept	-1.00	0.16	<.001	0.37	0.27-0.50
	Employment support	1.04	0.30	.001	2.82	1.56-5.08
2	Intercept	-1.01	1.98	.609	0.36	0.01-17.69
	Employment support	0.93	0.33	.005	2.54	1.32-4.89
	Long-term condition	-0.71	0.30	.020	0.49	0.27-0.89
	Problem descriptor	0.12	0.31	.708	1.12	0.61-2.06
	PHQ-9 baseline	0.02	0.06	.664	1.02	0.92-1.14
	GAD-7 baseline	-0.05	0.06	.397	0.95	0.85-1.07
	WSAS baseline	-0.04	0.03	.193	0.96	0.91-1.02
	Total sessions	0.13	0.03	<.001	1.14	1.08-1.20
	Gender	-0.18	0.32	.572	0.83	0.44-1.57
	Ethnicity	0.14	0.34	.689	1.15	0.59-2.23
	Age	0.02	0.01	.198	1.02	0.99-1.05

*Notes*: Overall results model 1: *χ*^2^ (1, n = 302) = 11.82, *p* = .001, *R*^2^ (Cox & Snell) = 0.04, *R*^2^ (Nagelkerke) = 0.06; Overall results model 2: *χ*^2^ (10, n = 302) = 46.04, *p* <.001, *R*^2^ (Cox & Snell) = 0.16, R^2^ (Nagelkerke) = 0.22

## Data Availability

The data that support the findings of this study are available on request from the corresponding author. The data are not publicly available due to privacy and ethical restrictions.
